# Educational psychology empowers the complete utilization of mobile medical education resources: strategies and practical proposals

**DOI:** 10.3389/fpsyg.2026.1786536

**Published:** 2026-06-05

**Authors:** Li Liu, Jiangfeng Li, Xiaochuan Wang, Fangfang Yu, Zhiguang Ping

**Affiliations:** 1 School of Basic Medical Sciences, Zhengzhou University, Zhengzhou, China; 2 School of Continuing Education, Zhengzhou University, Zhengzhou, China; 3 College of Public Health, Zhengzhou University, Zhengzhou, China

**Keywords:** educational psychology, mobile educational resources, mobile medical education, practical proposals, strategies

## 1 Introduction

Mobile medical education is progressively emerging as an innovative force in the domain of medical education. It offers flexible and convenient educational services or learning pathways to medical learners through online course platforms or specialized apps relying on mobile communication technology and smart portable devices, such as smartphones and tablet computers (Altintas and Sahiner, 2024). With the rapid development of the internet, digital intelligence technology, as well as the popularization of mobile communication equipment, mobile medical education has gained prominence since the early 21st century (Ogundiya et al., 2024). In recent years, particularly following the COVID-19 pandemic outbreak, global advancements in mobile medical education have been remarkable. Currently, it encompasses various forms including online courses, virtual simulation laboratories, remote internships, online assignments, online assessment, and so on, providing a broad range of learning opportunities and practical platforms for medical learners worldwide. This approach not only enriches the accessibility to medical education resources but also breaks the temporal and spatial constraints of traditional education models. Thus, high-quality medical education resources can be widely disseminated and shared while aiding students in adapting to and keeping pace with technological advancements (Walsh, 2015; Walton et al., 2005). As a result of these benefits, many medical colleges have incorporated it into their teaching system, and numerous medical continuing education projects also rely on mobile platforms (Curran et al., 2019; Rajasekaran and Iyengar, 2013).

While mobile medical education resources possess significant advantages, several challenges have emerged in current practice (Iqbal et al., 2020; Ishak et al., 2022). During the course construction, it is observed that the issue of high dropout rate among students is prominent, which seriously hampers teaching continuity and completeness, thereby hindering the achievement of educational objectives. Meanwhile, some high-quality mobile education resources experience low click rates, despite their potential to greatly facilitate student learning, and have not been fully appreciated and utilized (Iqbal et al., 2020). Consequently, this situation impedes the effectiveness of mobile medical education in stimulating student interest and achieving expected teaching effect.

The phenomenon that mobile medical resources are not fully exploited by students is worthy of reflection. Education is not merely the imparting of knowledge but primarily concerned with educating people (Zhang, 2015). At present, teachers hold high expectations for new technologies to facilitate teaching, while students show little interest in new technologies due to being accustomed to massive online resources. Therefore, teachers should have a profound understanding of the psychology of medical students, prioritize the fundamental concept of people-oriented. By incorporating educational psychology strategies into medical education, teachers are likely to guide students to actively, positively and reasonably use mobile medical resources to improve learning outcomes.

Educational psychology assumes a crucial role in medical education (Zhang, 2015). In the realm of mobile medical education, it enables teachers to comprehend the psychological and learning traits of medical students. By integrating medical educational psychology with new technologies, we can design efficient and scientific teaching approaches that align with the psychological requirements of medical students. Recent international studies have validated that educational psychology theories effectively enhance student engagement in digital medical learning environments (Hadie et al., 2021). For example, a qualitative study of 15 medical students from the University of Birmingham found that digital learning tools consistent with self-determination theory can successfully stimulate students' intrinsic learning motivation (Kulkarni et al., 2025).

This article will combine the principles of educational psychology and adhere to the general principle of being people-oriented, and propose several strategies to enhance mobile medical education, with the aim of assisting medical educators in making better utilization of advanced technology to improve the quality of medical education. At the same time, we also put forward corresponding implementation suggestions and concise implementation notes, enabling teachers to make flexible choices during the construction of specific mobile resources. Furthermore, the application of these strategies in resource-limited areas and diverse cultural contexts is also fully considered.

## 2 Strategies and practical proposals

### 2.1 Focus on the fundamentals of teaching while avoiding being over-obsessed with technology

Education is undoubtedly influenced by technology; however, its fundamental purpose is to cultivate people. Maintaining a favorable relationship between teachers and students and cultivating a harmonious learning atmosphere are the fundamental conditions for sustaining students' learning motivation (Mo, 2007). Although advanced technology offers convenience and support for education, it cannot supplant the core role of teachers (Zhang, 2015).

Currently, despite students having numerous electronic resources, their learning outcomes are unsatisfactory due to the lack of guidance. Therefore, in the era of significant innovation in the medical education model, educators should pay attention to the following points: a. Based on the actual situation of students, select teaching methods and technical means: utilize modern technology rationally to assist teaching and facilitate students' efficient learning and all-round development. Teachers' professional development project with teaching method as the core helps hospital school teachers to effectively integrate mobile technology into teaching, which significantly enhances teachers' confidence and teaching effect (Maor et al., 2016). b. Education should not be constrained by technology but return to its roots: take students as the center and let technology serve teaching. Focus on students' needs and the learning process, stimulate their interest in learning, cultivate their independent and innovative thinking, help them correct their attitudes and master methods, guide them to learn actively (Reefman et al., 2017). c. Pay attention to the cultivation of teachers' own basic teaching qualities, reflect on the teaching content and methods. Inspire and motivate students to actively explore medical resources with teachers' rigorous academic attitude. Excessively pursuing new technology and neglecting the fundamentals of teaching is putting the cart before the horse. Based on global online education research, Rosser-Majors M. L. demonstrated that instructor presence is a more significant predictor of online course completion rates than technological tools alone (Rosser-Majors et al., 2022). d. Guide students to have a correct understanding of new technologies, making it clear that mobile technology is only an auxiliary tool and cannot replace in-depth learning and thinking. e. The evaluation of teaching should not overly emphasize the indicators of new technology utilization but should focus on teaching effectiveness, students' needs and development, thereby avoiding medical education descending into technical formalism, and truly enhancing the teaching quality and benefiting students (Alshammari et al., 2024).

Implementation notes: this strategy requires core resources including basic teaching materials, student-centered pedagogical training or supporting documents, as well as mechanisms for teaching reflection and academic exchange. Potential challenges include educators' overemphasis on technological applications and inadequate teaching reflection, which can be mitigated through regular pedagogical training, simplified adoption of technical tools, and strengthened effectiveness-oriented teaching evaluation. Simple evaluation indicators consist of student learning satisfaction, effective utilization rate of mobile resources, teaching effectiveness scores, and teachers' teaching reflection reports.

### 2.2 Give students the autonomy to utilize mobile education resources

Students are one of the important subjects in educational activities. So satisfying students' needs for autonomy, competence, and relatedness is crucial for stimulating learning motivation (Zhang, 2015; Cleland and Durning, 2020). In the educational domain, this implies that students should have the right to make their own decisions regarding learning resources, so as to ensure that their learning motivation and autonomy can be fully exerted. Imposing technology upon students and disregarding their right to choose would violate the principles of educational psychology and lead to students' resistance to technology (Li et al., 2024).

To enhance students' intrinsic motivation for using mobile educational resources, educators may consider the following strategies: a. Provide diversified learning resources and permit students to select their own learning content and methods based on their own interests and learning needs, encourage students themselves to evaluate their learning effects, thus make students truly sense the subjectivity of learning and control the learning process and outcomes, and effectively prevent the rebellious psychology caused by the passive use of new technologies. Triebner et al. demonstrated that allowing students to choose and distribute their own learning tasks significantly enhanced their perceived autonomy and intrinsic motivation while reducing stress (Triebner et al., 2024). b. Establish an interactive platform such as a message area to encourage students to share ideas, ask questions, and discuss, which not only meets the relationship requirements but also promotes the deepening of knowledge. Teachers should pay attention to students' messages in time and adjust mobile education resources timely to ensure that learning resources match students' needs. c. The integration of artificial intelligence technology. Gupta et al. pointed out that generative AI can provide personalized learning experiences based on individual learners' needs, including customized study plans, targeted practice questions, and clinical cases, thereby meeting students' competence requirements and improving learning efficiency (Gupta et al., 2024).

In conclusion, it is an effective approach to stimulate students' intrinsic motivation and optimize the utilization of mobile education resources by giving students the right to make their own decisions and making technology serve their independent choices.

Implementation notes: this strategy requires core resources including a diversified library of mobile learning resources, personalized learning support tools, and teacher-student interaction platforms. Potential challenges include insufficient students' autonomous planning ability, blind selection of resources, and low participation in interaction, which can be mitigated by providing guidance for resource selection, and strengthening teachers' real-time guidance and feedback. Simple evaluation indicators include completion degree of autonomous learning, frequency of teacher-student interaction participation, and scores of learning intrinsic motivation.

### 2.3 Simulate practical situations to facilitate in-depth learning in medical settings

Situated cognition emphasizes that learning should be carried out in concrete, situational and perceptible activities to deepen students' comprehension of the application and value of knowledge (Zhang, 2015). In medical education, the application of this theory is anticipated to significantly enhance the learning outcome.

The specific implementation approaches include: a. Constructing a situational knowledge system, that is, linking relevant practical teaching contents when introducing medical knowledge points (Sha and Wang, 2019). For instance, in PPT, video explanations and knowledge map nodes of basic medical course modules, the related virtual anatomy experiments, histology digital slice libraries, virtual functional energy experiments and explanations can be linked. In the statistical method introduction part of the health statistics course module, practical application cases in published papers and actual operation videos of SPSS data analysis can be linked, enabling students to intuitively perceive the practicality of knowledge (Quinapanta Castro et al., 2026). b. Combine virtual reality technology with mobile devices such as PAD and smart phones to create an immersive learning environment. Wang et al. confirmed that combining large language models with mobile devices creates an immersive standardized patient environment for clinical skills training (Wang et al., 2025), while Dhar et al. further demonstrated that integrating VR technology with mobile devices such as HMDs and smartphones constructs highly immersive learning environments that significantly enhance knowledge acquisition, skill mastery, and learner confidence in medical education (Dhar et al., 2023).

Implementation notes: this strategy requires resources include virtual simulation software, a clinical case video library, and mobile VR devices (with simplified versions provided for resource-limited settings); potential challenges involve high equipment procurement costs and a high technical operation threshold, which can be addressed by adopting free and open-source virtual simulation tools and basic mobile devices or cooperating with professional digital technology institutions; evaluation indicators consist of students' scores in the objective structured clinical examination, satisfaction survey results, and the completion rate of operational tasks.

### 2.4 Guide students to explore actively through questions to coordinate cognition

Cognitive dissonance theory indicates that once cognitive dissonance occurs, an attitude change is needed to restore the state of cognitive harmony (Zhang, 2015). In mobile medical education, teachers can intentionally induce students' cognitive dissonance through teaching design to stimulate students' interest in active exploration.

**Figure 1:**
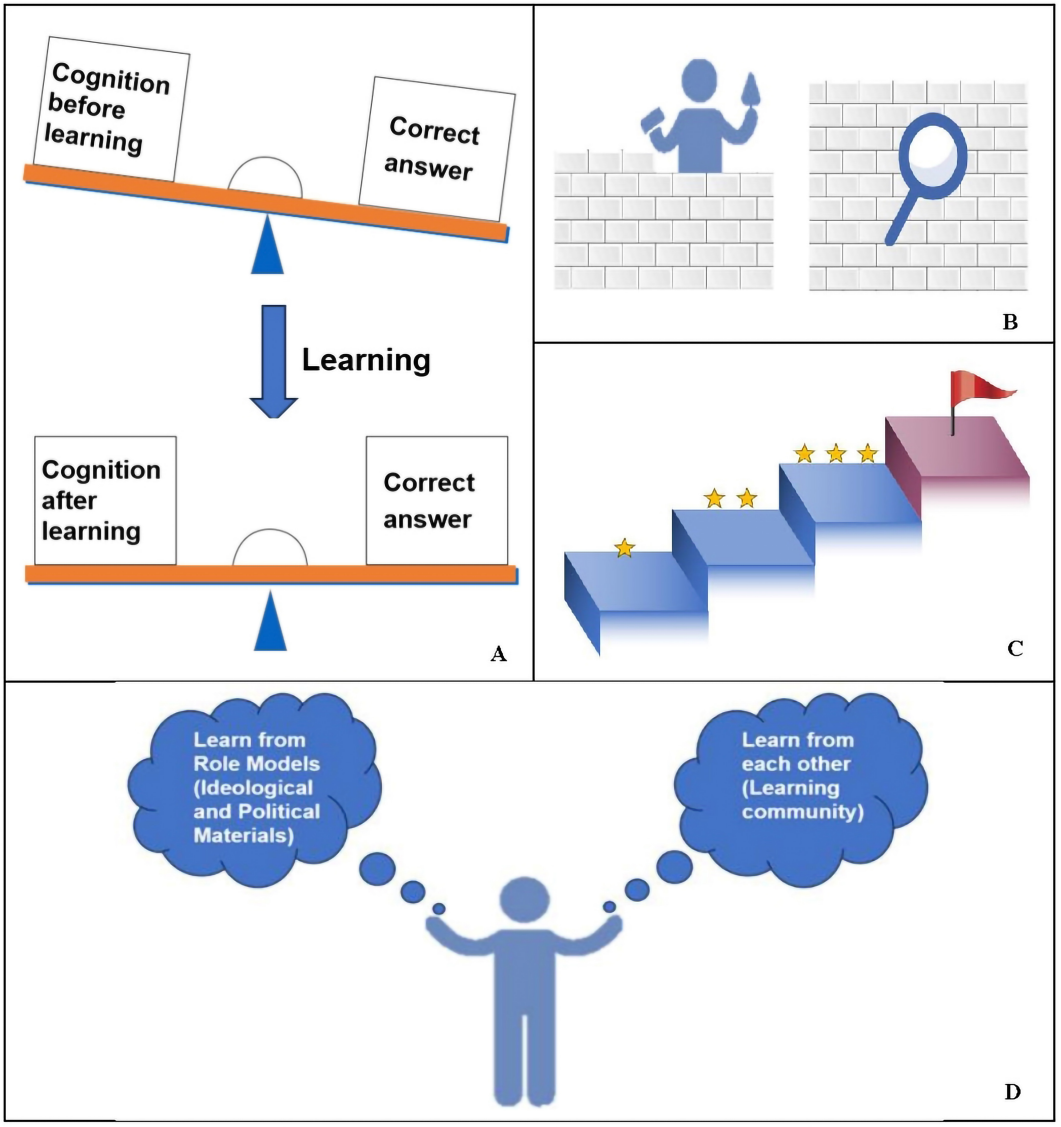
Some psychological strategies in mobile medical education. **(A)** Guide students to explore actively through questions to coordinate cognition. **(B)** Encourage active construction by interacting with students regarding mobile resources. Students participate in the construction of mobile resources and conduct error identification and correction. **(C)** Set graded tasks and incentives to enhance students' sense of self-efficacy. **(D)** Social learning strategies: students improve themselves through observation and imitation.

The specific strategies are as follows: before introducing the course knowledge points, the problems related to students' learning and life experiences but with cognitive conflicts can be embedded. For example, in the course module of embryology, the question “What parts of the human body will the primitive digestive tract develop into?” can be set before the chapter. Students may think that it will develop into an organ of the digestive tract based on experience. However, the knowledge map reveals that it is related to the organogenesis of the digestive tube, digestive gland and even the respiratory system. This kind of cognitive disparity will lead to students' sense of imbalance and impel them to actively seek answers. Then, students are guided to further explore the corresponding embryology content by consulting mobile medical resources or participating in online discussions. For another example, in the course module of medical statistics, you could set the question that “Is the ice cream sales related to the crime rate in summer?” to stimulate students' interest in thinking and guide them to learn the online course of the chapter “correlation,” so that they can deeply understand the difference between correlation and causality (Figure 1A).

When adopting the cognitive dissonance strategy, attention should be paid to the following: firstly, it is necessary to ensure that the questions are both close to students' reality and challenging, so as to guide students to discover cognitive differences. Additionally, students should be encouraged to actively explore and correct cognitive deviations. Kojima et al. found that adding a step requiring medical students to actively articulate “what does not fit” with their predicted diagnosis (i.e., verbalizing evidence that contradicts the diagnosis) to the traditional teaching method can help students further learn and reflect, effectively correcting cognitive biases (Kojima et al., 2025). To sum up, designing questions with cognitive dissonance theory and guiding students to actively explore mobile medical resources is an effective way to promote students' deep learning and improve learning results.

Implementation Notes: required resources include teacher training on how to design effective cognitive dissonance questions and the establishment of an online discussion platform. Potential challenges lie in designing questions with an appropriate difficulty level. Questions that are overly simple may fail to trigger cognitive dissonance, while those that are excessively difficult may cause student frustration. Mitigation strategies include piloting the questions among a small group of students before large-scale implementation and conducting iterative optimization based on student feedback. Evaluation can be carried out through pre- and post-question knowledge tests, analysis of forum participation quality, and surveys on students' course engagement.

### 2.5 Encourage active construction by interacting with students regarding mobile resources

From the perspective of the active construction theory in educational psychology, learning is a process of actively constructing new knowledge through interaction with the environment based on existing knowledge, that is, if students wish to acquire knowledge and skills, they must discover and transform complex information themselves (Slavin, 2024).

In the construction of mobile medical education resources, students' participation can significantly enhance their learning initiative and knowledge construction ability. Examples of possible approaches are as follows. a. Organize students to collaborate in groups to build mobile resources and share their achievements, thereby deepening their understanding and mastery of knowledge through cooperation and exchange. Floren et al. found that medical and pharmacy students, through asynchronous online collaboration to develop care plans for complex cases, significantly improved their clinical reasoning skills (Floren et al., 2020). In practice, due to the complexity of the construction of curriculum resources, some students with extra energy and capacity can be selected to participate in specific tasks on the premise of complete voluntariness, such as the structural annotation of virtual anatomical specimen or digital slices, and the linking of knowledge points in knowledge graph. b. Set up a message area or discussion area in the course construction to encourage students to identify mistakes in mobile education resources and put forward suggestions for improvement, which unconsciously prompts students to comprehensively examine the content of resources and promotes interaction among students, as well as between students and teacher, which is conducive to cultivating students' critical thinking and innovative ability (Figure 1B).

In conclusion, by employing the active construction strategy, students can not only make full use of mobile education resources but also stimulate their learning potential and cultivate their knowledge construction ability. However, when using this strategy, teachers should avoid forcefully implementing active construction, which may impose a burden on students lead to counterproductive teaching effect.

Implementation notes: the core resources required for this strategy include mobile educational resource development templates, online collaboration tools, interactive discussion and feedback platforms, and a resource review mechanism. Simplified versions of collaboration tools and online shared documents can be provided for resource-limited settings. Potential implementation challenges include students' limited time and energy, as well as uneven quality of self-constructed learning resources. Mitigation measures include clarifying the principle of voluntary participation, organizing simplified training for resource development, streamlining task design, implementing peer review of student contributions, and arranging ongoing faculty supervision. Evaluation indicators cover the quantity of student resource contributions, quality assessment of submitted resources, and the adoption rate of improvement suggestions.

### 2.6 Set graded tasks and incentives to enhance students' sense of self-efficacy

The theory of self-efficacy emphasizes an individual's confidence and belief in accomplishing a certain task (Zhang, 2015; Slavin, 2024). In medical education, self-efficacy has a significant impact on the learning autonomy motivation of medical students (Zhu et al., 2024). Examples of application methods are as follows. a. Design a graded task system in the course. For instance, set questions with varying difficulty levels: provide simple questions in the initial stage, enabling students to complete them easily and gradually build up confidence. Then, gradually increase the difficulty of the task, allowing students to clearly perceive the improvement of their own ability in the challenge, and can be competent for the learning task, thereby stimulating their enthusiasm for learning. b. Set up a positive incentive mechanism in the course. For example, offer timely and concrete recognition and praise to students' efforts and progress through points, medals, upgrades, and other forms. When students sense the rewards and value of hard work and the joy of growth brought by self-transcendence, their self-confidence and self-efficacy may be significantly strengthened. Long et al. effectively enhanced the academic self-efficacy of medical students with academic burnout through the pathway of positive reinforcement (Long and Long, 2025). It should be noted that teachers should design reasonable incentive mechanisms according to the specific situation and psychological needs of students to avoid boredom or childishness (Figure 1C).

In summary, measures such as designing graded tasks and implementing positive incentive mechanism can effectively improve the self-efficacy of medical students, encourage them to make full use of mobile resources, and improve their medical knowledge literacy and skills.

Implementation notes: required resources include a graded task bank, an incentive system, and a learning progress tracking module. Potential challenges involve unreasonable task difficulty configuration and insufficient appeal of incentive mechanisms, which can be addressed by dynamically adjusting task difficulty and investigating students' preferences for incentive formats. Evaluation can be performed by comparing academic outcomes between incentive and conventional teaching modules, monitoring the progression rate of hierarchical tasks, measuring satisfaction with the incentive scheme, and assessing students' self-efficacy.

### 2.7 Integrate ideological and political elements to inspire students to improve themselves through observation and imitation

Social learning theory emphasizes the role of observation and imitation in learning (Zhang, 2015; Slavin, 2024). In medical education, integrating ideological and political elements into mobile educational resources enables students to intuitively perceive the power of exemplars, thus being positively influenced and generating the motivation for imitation and learning (Yu and Zhang, 2022).

The following takes the construction of online courses as an example to introduce the concrete steps of integrating ideological and political elements. First, the integration of ideological and political resources should be designed systematically in the course. Then, these materials should be extensively collected: the development history and milestones of this discipline, the contributions and stories of outstanding scientists or doctors, relevant knowledge of traditional Chinese medicine, etc, Subsequently, according to the syllabus and chapter focus, the above contents will be accurately screened and matched. Finally, these contents will be linked into mobile resources in an appropriate form and place, such as establishing links in PPT or the knowledge graph.

The ideological and political elements play a significant role. It was found that after integrating ideological and political education into digital teaching resources, the experimental group students' comprehensive scores, moral identity, and learning engagement were significantly higher than those of the control group, confirming that embedding the power of exemplars into mobile resources can effectively stimulate students' motivation for imitative learning (Zheng et al., 2025). Additionally, an online learning community can be established in the course to encourage students to share their learning experiences, thereby creating a favorable atmosphere for active learning. A study found that the student-run social media learning community (WITI), through its decentralized and collaborative mode of interaction, effectively facilitated mutual imitation, peer motivation, and knowledge co-construction among students, thus significantly improving learning engagement (Han et al., 2025) (Figure 1D).

Implementation notes: required resources include a role model story database and an online learning community platform. Potential challenges involve poor compatibility between role model materials and teaching content, low emotional resonance among students, and insufficient interaction within the online learning community. Corresponding mitigation measures consist of selecting materials in combination with teaching contexts, adopting diversified role model stories, and innovating presentation forms such as short videos and illustrated texts to evoke resonance among all types of students. Evaluation indicators cover the utilization rate of role model materials, surveys on students' learning initiative and professional identity, and statistical data of community interaction.

The seven strategies proposed in this study have the potential to flexibly adapt to different global cultures and resource-constrained scenarios. In low-resource regions, practical implementation can be realized by adopting free open-source mobile data, simplifying resource libraries, prioritizing offline design, constructing peer assistance models based on existing social structures, and making maximum use of low-cost technologies such as basic smartphones and remote guidance. For example, Chepkoech et al. pointed out that remote guidance programs for low- and middle-income countries are expected to improve the accessibility of surgical education. The hybrid approach combining low-cost 5G deployment with satellite communication can guarantee reliable network connectivity in areas without fiber-optic infrastructure (Chepkoech et al., 2025). In terms of cross-cultural adaptation, the strategies can be aligned with local medical education norms and students' cultural backgrounds by adjusting case materials, role model stories and interactive forms, so as to ensure their global applicability.

## 3 Limitations and future research directions

This paper is a conceptual opinion article formed based on educational psychology theories and frontline teaching experience, and large-scale empirical verification has not yet been conducted. Although some strategies in the article have incorporated empirical cases, this study has not implemented systematic pilot studies, randomized controlled trials, or longitudinal observations by treating the seven strategies as a comprehensive intervention program. The overall application effect of the strategies still requires scientific validation. Future research may focus on the following directions: a. Establish a standardized evaluation system for strategy implementation to quantitatively assess student engagement, resource utilization, and learning effectiveness; b. Conduct cross-cultural and cross-resource setting comparative studies to examine the contextual adaptability of the strategies; c. Explore potential unintended consequences such as technology-induced anxiety and digital fatigue and develop mitigation programs; d. Explore the integration pathways of emerging technologies such as generative artificial intelligence into the framework of this study.

To sum up, the auxiliary status of mobile learning technology in medical education cannot be disregarded. Its integration with the traditional teaching mode has become a significant topic in the reform of medical education in the new era. To address the challenges of student engagement and mental health brought about by this change, this paper proposes to utilize the power of medical education psychology to optimize resource allocation and innovate educational models. Our aim is establishing a mobile medical education system that can not only efficiently convey medical knowledge but also take into account the healthy development of students' mental health and promote their active learning. The strategies and implementation suggestions mentioned in this paper are merely preliminary notions, and their effectiveness and feasibility need to be deeply explored, objectively evaluated, adjusted and optimized by medical educators in practice.
